# Complete Genome Sequence of *Alphaproteobacteria* Bacterium Strain SO-S41, Isolated from Forest Soil

**DOI:** 10.1128/MRA.00536-21

**Published:** 2021-07-22

**Authors:** Motoyuki Watanabe, Kensuke Igarashi, Souichiro Kato, Yoichi Kamagata, Wataru Kitagawa

**Affiliations:** a Graduate School of Agriculture, Hokkaido University, Sapporo, Hokkaido, Japan; b Bioproduction Research Institute, National Institute of Advanced Industrial Science and Technology (AIST), Sapporo, Hokkaido, Japan; c Bioproduction Research Institute, National Institute of Advanced Industrial Science and Technology (AIST), Tsukuba, Ibaraki, Japan; Montana State University

## Abstract

The complete genome of hydrogen peroxide-sensitive alphaproteobacterial strain SO-S41 was sequenced. The complete genome contains a single chromosome, is 4,443,179 bp in length, contains a total of 4,632 genes, and has a G+C content of 66.2%.

## ANNOUNCEMENT

The high hydrogen peroxide (H_2_O_2_) level generated during agar medium preparation decreases the total colony count of inoculated environmental microorganisms (1–3). The strain SO-S41, which is highly sensitive to H_2_O_2_ was exclusively cultivated on an agar medium with a diminished amount of H_2_O_2_. Even if the medium is used, this slow-growing bacterium requires more than 1 week to form observable tiny colonies (1). This strain was isolated from the soil in the forest belonging to Hokkaido University, Sapporo, Hokkaido, Japan, and our previous study indicated that it might be phylogenetically novel at the order level (1). Here, the complete genome sequence of the strain SO-S41 is reported in order to obtain further understanding of its physiology.

The strain SO-S41 was grown in peptone-yeast-glucose (PYG) liquid medium (2) for 2 weeks at 20°C, and then its genomic DNA was extracted using the phenol-chloroform method (4). Short-read sequencing was performed using an iSeq 100 platform with paired-end 150-bp reads, and the sequencing library was prepared with a Nextera DNA Flex library prep kit (Illumina). The reads were trimmed with Trimmomatic v0.39 (SLIDINGWINDOW:6:30 MINLEN:78, and other parameters at the defaults) (5). In total, 923,927 paired-end and 167,116 single-end reads were obtained.

Long-read sequencing was performed using a MinION Mk1B platform, and the sequencing library was prepared with an SQK-RAD004 kit (Oxford Nanopore Technologies). The reads were base-called using the high-accuracy mode of MinKNOW v4.1.22 and then trimmed with Nanofilt v2.7.1 (- -quality 10, - -length 500, - -headcrop 75, and other parameters at the defaults) (6), and the quality was assessed with NanoStat v1.5.0 (6). In total, 20,906 reads (*N*_50_ read length of 1,350 bases, total read length of 25,855,567 bases) were obtained.

The hybrid assembly of iSeq and MinION reads was performed using SPAdes v3.14.1. (-k 21,33,41,55,77, - -nanopore, - -isolate, and other parameters at the defaults) (7, 8). Both paired-end and single-end reads from the iSeq sequencing were utilized in the hybrid assembly. As a result, three contigs which are longer than 200 nucleotides (nt) were generated (lengths of 3,781,019 nt, 600,517 nt, and 55,596 nt; coverages of 30×, 29×, and 29×). Outward-directing primers were designed from the ends of contigs and used in the PCR, using genomic DNA as the template. Amplification confirmed that three contigs are parts of the single circular chromosome. The region between contigs was determined by sequencing amplicons using an Applied Biosystems 3500xL genetic analyzer (Thermo Fisher Scientific). The assembled circular chromosome was annotated using Prokka v1.14.6 (9) with default parameters. The genome was rotated manually to the start of the *dnaA* gene.

The genome consists of a single chromosome of 4,443,179 bp with a 66.2% G+C content. The chromosome contains a total of 4,362 genes, 4,304 protein-coding genes, 53 tRNA genes, 4 rRNA genes, and 1 transfer-messenger RNA (tmRNA) gene. The genome contains major H_2_O_2_ scavenging enzyme genes such as two catalase genes and two alkyl hydroperoxide reductase genes.

### Data availability.

This whole-genome sequence has been deposited in DDBJ under the accession no. AP024629. The raw sequence reads have been deposited in the DRA under accession no. DRA011975.
